# Co-creating an antenatal care information resource in Arabic with re-settled migrant mothers

**DOI:** 10.3389/fgwh.2026.1717211

**Published:** 2026-02-20

**Authors:** Mabel L. S. Lie, Caroline Claisse

**Affiliations:** 1 Population Health Sciences Institute, Newcastle University, Newcastle upon Tyne, United Kingdom; 2 Open Lab, School of Computing, Newcastle University, Newcastle upon Tyne, United Kingdom

**Keywords:** antenatal care, Arabic-speaking women, co-creation, community engagement, participatory research

## Abstract

**Introduction:**

Arabic-speaking women newly settled in the UK have different experiences of healthcare in their original countries which influence their engagement with the NHS. Like other migrant women, they are often unaccustomed to the way health services work. Language, religious and cultural barriers, as well as the lack of family and community supports contribute to inequalities in health and wellbeing outcomes during pregnancy and childbirth.

**Materials and methods:**

To address their needs, the ERicar2 project aimed to produce a co-created antenatal care community information resource with a group of these women. Ten participatory workshops over eleven weeks were conducted to listen to the women's stories of their pregnancy journeys, explore publicly available resources, and work together to produce a prototype of an information resource tailored to their community needs.

**Results:**

The qualitative findings from the workshops uncovered their experiences of miscarriage, their health seeking behavior, the role of religious belief and digital media, and the importance to them of their reproductive rights. The messages that they had for a newly arrived Arabic speaking woman were incorporated in a letter illustrated with their drawings, addressed to a fictional character “Dila” and containing QR codes to internet sites for pregnancy support. The letter was digitally animated and recorded in English and Arabic by volunteers among the research participants. The ERicar2 online resource is available for use by UK health providers and Arabic-speaking communities.

**Discussion:**

Engagement with migrant mothers through participatory co-creative workshops (ERicar) is a model that can be replicated. In a safe community space, women sharing their pregnancy journeys have led to mutual support and research insights for maternal healthcare. Employing bricolage and iterative co-creation methods, women have been able to contribute to an antenatal resource tailored to the needs of an expectant mother recently resettled in the UK.

## 1 Introduction

Birth preparedness through antenatal care promotes safe motherhood and a reduction in adverse pregnancy and childbirth outcomes through antenatal screening and surveillance (1). However, evidence of poorer prenatal health utilization by non-western women in highly industrialized countries has highlighted barriers to accessing antenatal care (2, 3). Antenatal screening and diagnosis pathways, and pregnancy outcomes may be complex, and confusing for many migrant women, who lack information in their own languages. In addition, interpreter miscommunication and concerns around confidentiality pose barriers to migrant mothers seeking to access care (4–6). Studies have shown that migrant women have experienced fewer opportunities to express their preferences, participate in decision-making, and to communicate well with care staff (4).

ERicar was the mnemonic adopted for the research project Engaging Roma women in the co-creation of an antenatal care information resource (7, 8). ERicar was successful in conducting workshops with a group of Czech Slovak Roma women, collecting their pregnancy stories, distilling the most important themes, and gathering the messages of advice that the women would like to convey to a newly pregnant Roma woman in the UK. This led to the co-creation of a zine in English and Czech, filled with these messages, illustrated with the collages created by the women, and supplemented with QR codes to NHS endorsed websites. The zine was developed as a printable resource, digitally available to the Roma community and to health providers, acting as a bridge or means of engagement with the community. Following on from the success of ERicar, the research team consulted with the NHS local maternity service providers about the potential for ERicar to be replicated with other newly settled community groups. On their advice, the ERicar project was carried out among Arabic-speaking women, hence the title “Engaging Resettled migrant women” to replace “Engaging Roma women” with the aim of including other groups of mothers.

According to the UK Office for National Statistics Census 2021, Arabic was found to be the most common language spoken among immigrants in Newcastle and Gateshead, which gave researchers a strong rationale to work with Arabic-speakers for ERicar2. However, the heterogeneity of this population group needs to be acknowledged, as Arabic-speakers with a range of migration histories have arrived from many different countries in the Middle East and North Africa. Among the newly settled, it was observed that there were higher rates of adolescent pregnancies among Syrian war refugees (9). A high incidence of post-traumatic stress disorder among Syrian immigrant adolescent mothers in another study underlined the importance of a positive childbirth experience for these women (10). During the Covid-19 pandemic, the lack of informal supports for resettled refugee women from Arabic-speaking countries was found to be particularly significant especially affecting their postnatal care (11). Systemic barriers faced by resettled Syrian women have been reported, including inadequate interpreter support and paternalistic healthcare provision (12). A study (13) based in an emergency department found that women generally preferred a female health professional specifically for their reproductive healthcare. Assumptions by healthcare providers about the women's Muslim identity and fears for loss of confidentiality was also found in these women's accounts of their healthcare experiences. Attempts at understanding Arabic-speaking women's experiences of maternity care have revealed their preference for natural remedies, indicating their need for counselling on evidence-based medical interventions during pregnancy (14).

Arabic-speaking women resettle in Western industrialized countries with a variety of language proficiencies and life skills that need to be taken into consideration (15). Language and communication issues were explored in a number of studies with Arabic-speaking mothers in European healthcare contexts. In Sweden, it was found that the idea of interpreters as neutral and detached needed to be challenged, and that there were ways for knowledge claims of the migrant mother to be negotiated in interactions with health professionals (16). Sources of healthcare knowledge varied according to age, and between different generations, with older women depending on Arabic sources and communities, while their daughters were influenced by hybrid (Arabic and Danish) and digital sources of information (17).

A number of studies reported on interventions to improve the maternal health and wellbeing of Arabic-speaking migrant women. A study in Australia (18) investigated the role of doulas and found that personal characteristics that instilled trust were more important than cultural matching for the Arabic-speaking women in their sample. Another study in Germany (19) involved the use of peer support, which was found to be lacking among these women (12). The peer-based health promotion intervention was facilitated through social media, making the case for more research into mobile devices supporting migrant health. In Sweden, there were positive moves towards the development of digital tools to support Arabic-speaking women in their pregnancies. A tablet application (20) to improve their communication at antenatal care appointments was found to be effective particularly in supporting dialogue. However, whether it was best used together with or without an interpreter was dependent on language ability. There were however several challenges to overcome as the researchers sought to develop culturally appropriate, usable and reliable content for the application (21). These included stakeholder collaboration in content creation that met obstetric guidelines while being sensitive to the users' cultural preferences. Finally, pregnancy apps have been the focus in two projects in Sweden, namely, a bespoke app for Arabic-speaking migrant women undergoing field testing (22) and an existing pregnancy app translated and adapted for use with Arabic and Somali speakers. This was found to be helpful for health professionals in supporting healthy lifestyle behaviours (23).

## 2 Study design

### 2.1 Aims and objectives

In replicating the work of ERicar, the aim of the ERicar2 project was to co-create a “product” conveying key messages about antenatal care that could be scaled up as created outputs with digital components. The objectives of ERicar2 were to:

Explore with a group of Arabic-speaking women, their experiences of healthcare services and cultural beliefs about pregnancy and childbirth
Work with them to assess the accessibility and acceptability of a range of publicly available antenatal information resources
Explore and discuss options for forms of antenatal care information for example digital resources catering specifically to their needs
Co-create a low-resolution paper prototype of a community-based antenatal care information resource that meets their needs

A partnership with the Community Interest Company (CIC) that started with ERicar was further developed through an Arabic-speaking community coordinator and CIC administrative facilitator who worked together and recruited women from the “Tavga” women's network supported by the CIC. “Tavga” runs social activities for women from the Arabic and Kurdish communities in the North East of England organized through WhatsApp and Facebook. During the project, the research team drew on the expertise of advisors consisting of representatives from academia, the healthcare profession and the voluntary sector. The project was reviewed by the Ethics Committee of the Faculty of Science, Agriculture and Engineering, Newcastle University, on 28th August 2024 (24-030-CLA).

### 2.2 Methods

#### 2.2.1 Sample description

Seven of the ten women who were recruited were Kurdish, from Iraq, Syria and Iran, and three were Arabs. They arrived in the UK between 2010 and 2019. Most had lived in the UK from 5 to 9 years, with one mother who had resided in the UK for 14 years. They were aged between 22 and 64, and the ages of their children ranged from two to 24 years. Among them, the women had between one to six pregnancies, and seven women had experienced miscarriages. Five of the mothers had been cared for in UK hospitals, with four in Gateshead and one in Newcastle upon Tyne. Others were looked after in Syria, Iraq and Libya.

The characteristics of the sample are summarized in the Table 1 below:

**Table 1:** Sample characteristics.

Pseudonym	Age group	No of live births	Years in the UK	UK pregnancy	Years since last pregnancy
Khalida	45–54	4	6	No	17
Narin	45–54	4	5	No	22
Parveen	35–44	1	8	Yes	5
Aisha	35–44	2	5	No	8
Nourhan	15–24	1	8	Yes	2
Ghada	35–44	1	14	Yes	3
Fatem	55–64	3	7	No	30
Soleen	25–34	2	7	Yes	4
Laila	35–44	4	9	Yes	5
Maha	35–44	4	8	No	11

The participatory research method that was employed was dependent on accommodating the different language abilities of the women and the provision of interpretation for the purposes of mutual understanding in the workshops. Initially, an interpreter assisted in the workshops, but part way through, was unable to continue because of work demands, so the community coordinator stepped in. Arabic was the main language used among all the women except for one, who was conversant in English, Farsi and Kurdish. One mother was not literate in Arabic. Several women had varying levels of competency in spoken and written English and preferred to make their contributions to discussions in English rather than with the help of interpretation. Two women were confident enough to volunteer to interpret for others in the group. As the workshops progressed, discussions were found to work best with a mix of English and women interpreting for each other, and with the help of Google Translate.

#### 2.2.2 Workshops

The first workshop involved sharing the work of ERicar, distributing the translated information sheets^1^, explaining the ERicar2 project, obtaining consent and agreeing ground rules. The subsequent workshops followed the structure and format of the workshops in ERicar but included an additional workshop for co-creation of the resource. Table 2 summarizes the workshop activities:

**Table 2:** Workshop schedule.

Workshop	Date (2024–25)	Topic
1	11th Sept	Introduction, information and consent
2	18th Sept	Months 1–3 e.g., finding out you're pregnant, sharing news, 1st appointment
3	25th Sept	Months 4–6 e.g., keeping healthy and well
4	2nd Oct	Months 7–9 e.g., preparing for baby
5	9th Oct	Summary of pregnancy journey with artwork
6	16th Oct	Online antenatal resources from NHS and charities
7	23rd Oct	Pregnancy apps
		SCHOOL HALF-TERM BREAK
8	6th Nov	Exploring ideas for a new resource
9	13th Nov	Co-creating a new resource for Arabic-speaking women
10	20th Nov	Co-creating a new resource for Arabic-speaking women
Debrief	15th Jan	Feedback and planning for community event

**Figure 1:**
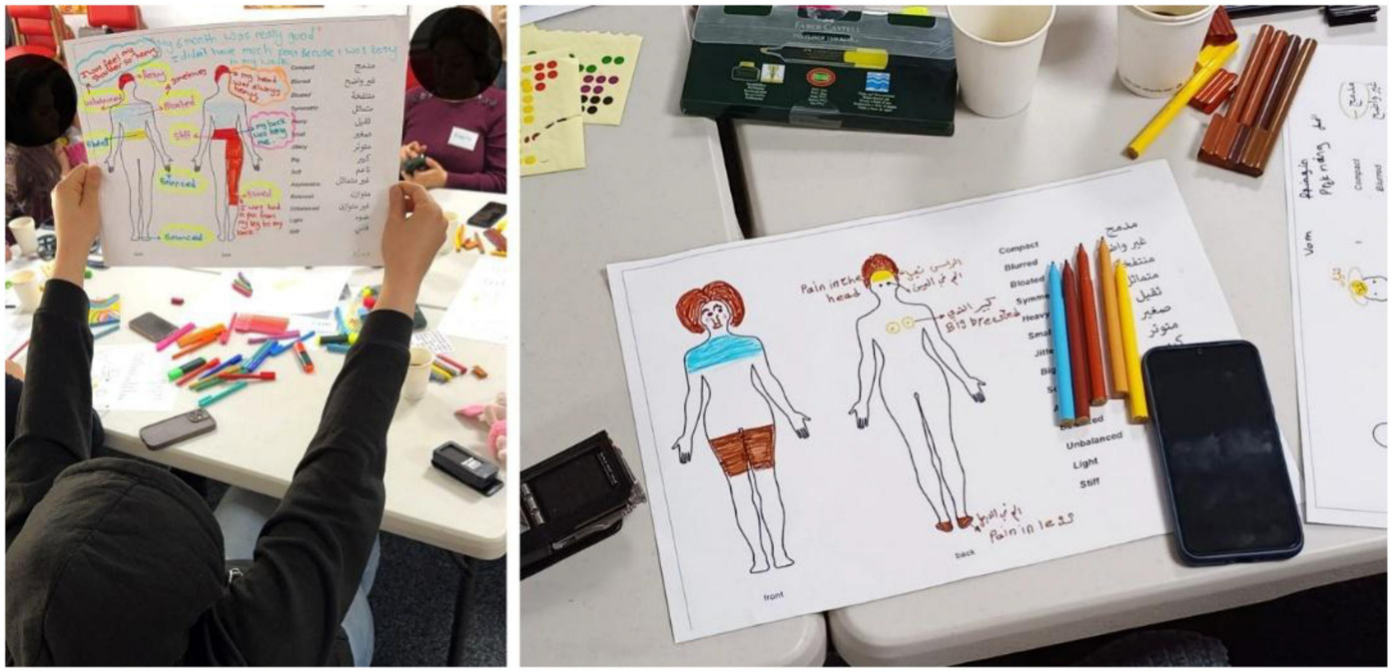
Body-mapping exercise, workshop 4.

*Workshops 2–5:* Storytelling of their pregnancy journeys covered issues such as access and experiences of healthcare, cultural beliefs about pregnancy and childbirth, language support, home remedies, family support, diet and exercise during pregnancy, and pregnancy-related health problems. The discussion that followed workshop 4 was scaffolded by means of a body-mapping exercise (24) (Figure 1 below).

**Figure 2:**
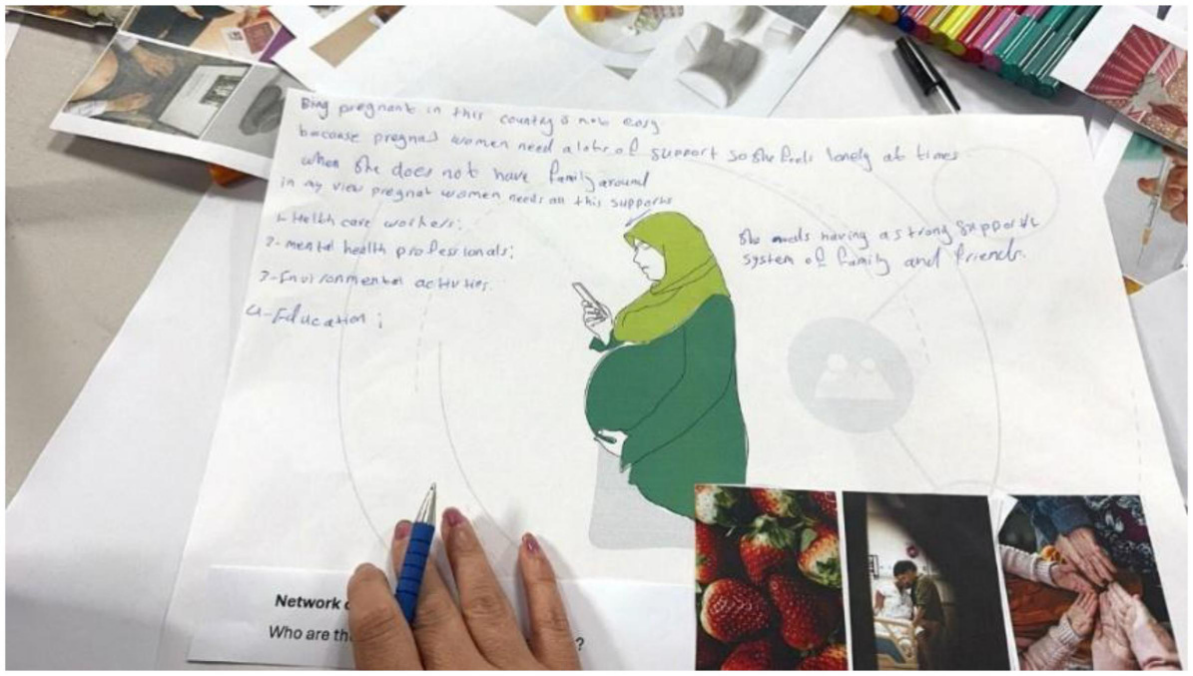
One woman mapping Dila's support network of care.

The women were encouraged to colour-in areas in an outline representing their body to express their awareness of pain or discomfort during their pregnancy. This was then used as a visual reference for sharing experiences during the workshop. In the fifth workshop, a summary of the findings from the three trimesters was presented to the women through a reading of the text paragraph by paragraph interpreted consecutively into Arabic for the women. Pauses between paragraphs provided an opportunity to reflect on what was said, to identify themselves in the anonymized narrative, and comment on what was said. This led to some revisions and validated the narrative. Inspired by participants' stories, a fictional character “Dila” as a newly arrived pregnant Arabic-speaking woman, was created and used to stimulate further discussion. Asked whether they could identify with Dila, participants were invited to create their own collage using cut-outs provided to support their comments (Figure 2).

**Figure 3:**
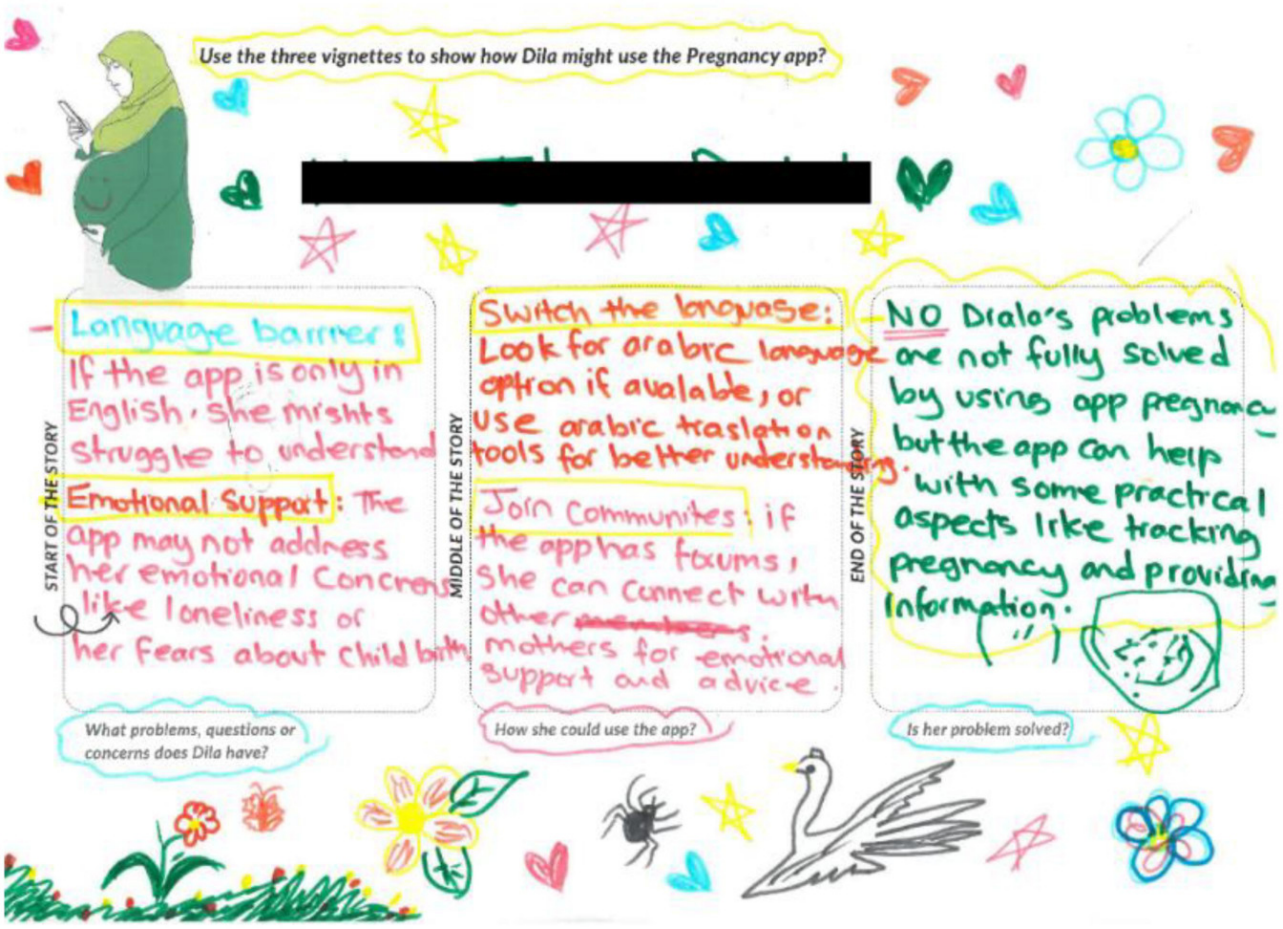
Activity sheet that prompted participants to consider how Dila might use the pregnancy apps and what challenges might arise.

*Workshops 6–7:* Different types and formats of various publicly available digital information resources were presented to the women. These composed of updated websites providing baseline antenatal information endorsed by the NHS such as NHS Start for Life, National Childbirth Trust, and the charities “Tommy's” and “Healthworks”. Textual and audio-visual forms of accessible antenatal support and information and key antenatal care topics were covered by these resources. These were presented in a chart for the women to indicate their strength of interest. In the next workshop, the women were presented with screenshots of existing pregnancy apps using PowerPoint and introduced to different features with the goal of identifying opportunities and challenges for women like Dila (Figure 3 below). Issues around the use of pregnancy apps, including privacy and security, and online community support were explored.

**Figure 4:**
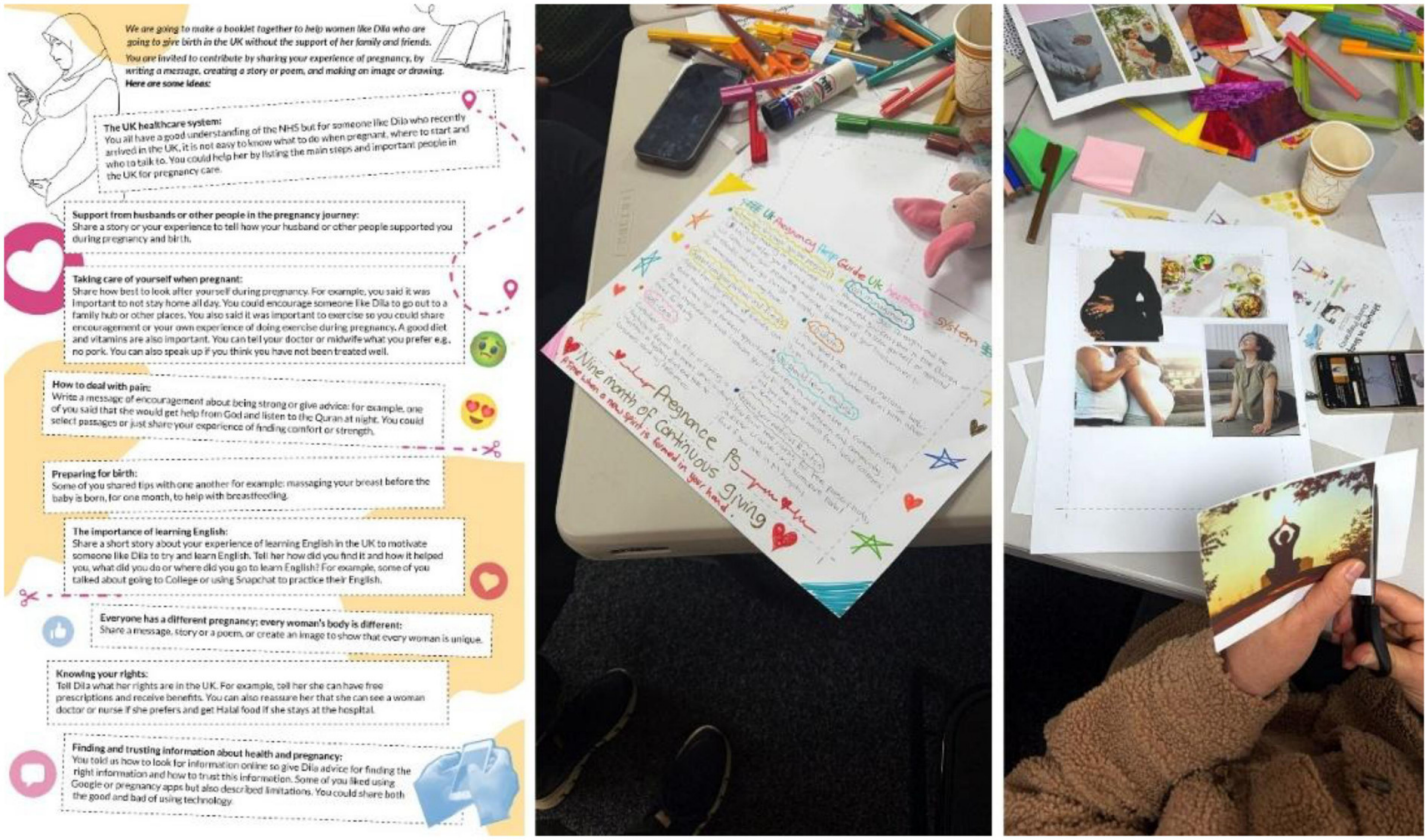
Activity sheet prompting women to contribute to a resource (left) and participants responding using drawings and images to communicate key message for the resource.

*Workshop 8–10:* A discussion on the findings from all the workshops was aimed at decisions with the women on the content and design of the information resource. This incorporated personal advice and what women learnt from each other's pregnancy experiences. A range of methods was shared, including storyboarding and zine/booklet-making that they could use to present the key messages they would like to convey to someone like Dila (Figure 4 below). In preparation for workshop 9, the women were invited to contribute images that could be used in an antenatal care information resource. Women worked in small groups to develop their own ideas and helpful messages to Dila. In the last workshop, drawing on all the women's contributions, thoughts and ideas, a “letter to Dila” was composed, with pictures from the women, and QR codes to relevant websites. This was recorded in Arabic and animated as an audio-visual digital resource.^2^

#### 2.2.3 Data collection and analysis

Factors that facilitated engagement and participation were 1) our community coordinator and administrative facilitator, who were known and trusted among the women, 2) the provision of shopping vouchers to thank women and 3) the relaxed atmosphere that allowed the women (and at times, children) the comfort of participating in discussions in a familiar environment. Conversations with the coordinator and facilitator revealed the effort and time invested, to recruit the women through reassurances to their husbands. Convincing the husbands of the value of the research was based on the CIC's reputation and the success of the ERicar project. The advisory panel provided a sounding board, and one of the advisors, i.e., the community midwife, took time to attend one of the workshops during refreshments to address women's concerns about their care. This further strengthened engagement with the women.

The authors are both experienced in qualitative research, ML in topics of maternal health and CC in co-design methods. We adopted a community-centred approach that aimed at giving voice to the women in a non-hierarchical safe space, especially important in underrepresented communities (25). Meetings with the advisory panel enabled reflections on the research process and emerging findings (26). Recordings were transcribed, anonymized, and entered into NVIVO qualitative data management software (27). The textual data was analysed thematically through systematic indexing, cross-referencing and retrieval in NVIVO. This led to the summary report written in accessible English, which was translated and validated by participants. Notes from participant observation as well as feedback about the workshops during the debriefing session were later included in the analysis, contributing to opportunities for iterative cross checking. Textual, audio and visual data was stored on MS Teams on a secure password protected university server and paper copies in secure university offices. The inductive thematic analysis conducted by ML produced 31 codes, which were then grouped into three themes for the purpose of this paper, namely, The Effects of Migration, Pregnancy Experiences, and Information and Communication.

Findings from these themes are followed by a discussion of the co-creation processes that were employed, which was a combination of the ideas of iterative co-creation and “bricolage”. Iterative co-creation is a cyclical design process used in knowledge translation that is based on open and honest collaboration, two-way learning and meaning-making, with the aim of refining the product (28–30). Bricolage is term coined by Levi-Strauss, to describe the “wild thinking” by researchers acting like “bricoleurs” or craftsmen using materials/resources at hand to create something innovative (31, 32). It involves employing a variety of skills using different tools imaginatively, e.g., visuals, structured or spontaneous interviews and informal knowledge, and allowing participants the use of a variety of linguistic and cognitive resources such as Google Translate. Bricolage is about employing an interpretive perspective of research carried out in the real world, not bound by rigid approaches.

## 3 Findings

### 3.1 The effects of migration

#### 3.1.1 Migrant identity, connections and faith

Most of the women in ERicar2 were part of the Kurdish diaspora from Iraq, Syria and Iran and three were Arabs from Syria and Iraq. The majority had refugee backgrounds having fled conflict situations in Iraq and Syria. As refugees, a number experienced resettlement several times, for example, moving from Iraq to Syria or from Syria to Lebanon before arriving in the UK. One woman described living in different parts of the UK before settling in the North East. While language can be a means of individual identity, a variety of Kurdish languages were spoken among them with degrees of fluency, e.g., Kurmanji in Syria, Badini and Sorani, spoken in Iraqi Kurdistan.

Of the three Arab women, one described herself as Assyrian Christian while the rest identified themselves as Kurdish. Nabiha, our community coordinator, added that in form filling for identification purposes, they would select “Arabic” because of the limited categories available, also because of their history of Arabization in their countries of origin. Ghada has on occasion selected “Other Asian” “because Iraq, there is no Middle East, we are from Middle East, is part of Asia, so we have to say Other Asian. It's not nice but  … very vague”. Soleen would sometimes identify as White or Asian but would type in “Arab” or “Kurdish” if she had a choice. Nourhan and Parveen would “prefer not to say”. Nevertheless, the women could also see the value of disclosing their ethnicity in certain circumstances, as clinicians might recognise common health problems faced by people in their part of the world. Disclosing their religion could also help explain their preference for female health professionals.

However, racial profiling can be problematic, affecting the doctor-patient relationship and confidence in NHS services:

Because once, I had on my face, I went to doctor, that doctor didn't do ANYTHING for me. He said, any Asian people come to this country, they have this problem, the skin goes darker. I said I am not from Pakistan, I know what you mean, I'm from Iraq. So yeah. (Ghada)

Discussions about women's relationships with other migrants revealed the caution they had to exercise in forming friendships:

it's really hard to make friends in this country because first of all you have to trust that person and you don't know them, what's the background, what's where they are came from, which family, which situation they live, no it's not easy to make friends. (Nourhan)

Ghada agreed, “you have to know them first  … some people you can't trust very easily”. Nourhan however disclosed that like Laila, she had friends from Africa, India and Pakistan.

The women were asked about their experience of being a Muslim woman in the UK and the wearing of the hijab which marked them out as different. Nourhan struggled with this because of not wanting to be judged at first sight on how she looked. Her perspective was reinforced in another's experience:

She said sometimes I see it in my eyes, when they are seeing a hijab girl, they just try to be back from her, and don't like, come to her or touch her. (Nourhan interpreting)

As a young woman, Nourhan was thankful her family supported her decision whether to wear the hijab which reflected the changes she witnessed as a migrant in this country. Regardless of her decisions about how to dress, Nourhan's religious faith played a part in coping with life in a different country.

I wasn't really big on my religion, but after that, when I start reading, the Koran, I feel more calm, I feel more happy.

The workshops were carried out shortly after the race riots that erupted after the Southport stabbings in July 2024^3^, and there was some feeling that British culture was changing. Nevertheless, the women felt that the wearing of the hijab need not feature in the antenatal resource they were creating. Religion however played an important role when women were in distress and in need of comfort in their pregnancy journeys:

she just pray, say to God please help me to get this baby out from me and put your hand on me. And then her daughter just came out. (Interpreter for Narin, Christian)

When the women viewed the antenatal information online, the women were very surprised to read that mass cremation for aborted foetuses was practised in this country. This was based on their Islamic religious beliefs which disallowed cremation. According to the women, fasting is not required during pregnancy, but some women still fasted in the early stages, assured that it would not affect the baby. Halal food was requested when they were in hospital, but some like Ghada simply asked not to have pork.

The discussions about migrant identity pointed to the limitations of official categories describing demographic characteristics. The challenges faced in who they could trust could translate to relationships with health professionals. Some women had developed positive relationships with the different people they met but would prefer to rely on trusted contacts, and their faith in God. Although the women had varied migration histories, in sharing their experiences, they found a common bond between each other, as coming from the Middle East, as migrant mothers settling in the UK, and as women of faith.

#### 3.1.2 Being supported, feeling alone

While migration experiences featured several times in incidental ways in the women's accounts, the women were not prompted to elaborate on their experience directly, because of the possible trauma attached. Life in their countries of origin was very different to being in the UK. The older women in the group described the support of relatives and the community:

Her husband and her sister they were supporting her so much. Her sister before she had 5 daughters before and three boys, so she got all the experience. (Interpreter for Fatem)

The women also described personal support from mothers-in-law, including relatives living abroad. In one case, advice given by a relative to avoid animal protein during pregnancy was accepted without question by Ghada, because to her, she was a trusted source of information.

When the discussion about the role of husbands arose, several women referred to their husbands being at work, sometimes working two jobs, to support the family. Kurdish migrant men are known to work in garages, car washes and other auto services.

My husband sometimes good sometimes no. I don't know why, both of them, when I bring the baby, husband's car is broken. 12th Dec was very cold, it was snowing, my husband's car is broken, he's in the service in the evening mechanic for car, yah, terrible. (Soleen)

Despite the women's varied migration histories and their use of social media, one theme that recurred was the experience of “being alone”, which was especially difficult when one was pregnant and feeling ill, as well as having to care for another child during Covid.

when I'm pregnant I got a cold flu, very bad [Covid], days when I can't move in a sofa and um no one around me just my husband he went to the work, and I have a very bad experience …. , it was lockdown, I didn't see anyone, just two kids, every time crying, lonely (Soleen)

Even without being ill or having another child to care for, a migrant mother on her own had to manage the pregnancy and the pain of labour when the time came to give birth.

I give birth I was by myself, I had no one around me. [Your midwife?] No, no, no, because I had labour pains at night, I thought kidney pain or something else, but I couldn't move, in the house by myself, and just call an ambulance, and they came straightaway (Parveen)

The thought of not having loved ones around to support especially before help arrived was a foremost concern among the women. The contrast between having family and friendship supportive networks and being left to fend for oneself emerged as a pertinent aspect of being a migrant mother in a foreign environment. This has implications for the perinatal mental health of women and the wellbeing of both mothers and their babies.

#### 3.1.3 Non-UK and UK healthcare

Some women had the experience of being pregnant and giving birth in their countries of origin or resettlement outside the UK. Narin described a traditional village midwife in Iraq coming to her house and the unsatisfactory care she received. But in a later pregnancy, she was very impressed with the doctor who looked after her when she moved to Libya as a refugee:

The doctor was very nice, very beautiful, in Libya. She said to the doctor that she was happy to see you. She was beautiful and she lit up the place. (Interpreter for Narin)

Fatem opted to go into the hospital for her second child where a doctor took special care of her because of her history of miscarriage. He continued to look after her when she had her other children.

In Syria, Laila had to pay for all her maternity care, unlike in the UK. She explained how the systems were different, where maternity care was on demand and paid for, whereas there was an established maternity pathway in the UK that was followed.

I tell the GP that I'm pregnant, they straightaway in my file, that you are pregnant, they make a lot of appointments for you to go to for blood test, for urine test, [here] here, yes in the GP or go to hospital, so they always care about you and the baby, until you give birth. In Syria, no, you have to. You have to pay money and you have to ask for yourself.

Most of the women were unaware of the maternity exemption certificate which entitled pregnant women to free prescriptions regardless of their employment or residency status. Soleen being on a spouse visa, spoke about having to pay the immigration healthcare surcharge including her prescriptions.

Sadly, Ghada described attending an appointment at a local NHS Trust where the healthcare professional thought that she was pregnant, without knowing that a miscarriage had occurred. This was despite Ghada having been hospitalized.

I went for my appointment, to a midwife. They were trying to write something and asked me about pregnancy, I said excuse me, I'm not pregnant now. They said what? We have this appointment because you are pregnant. I say no, my baby's gone. I start crying again. That was one very bad thing. Just stop it I want to forget it!

Aisha suffered several miscarriages in the past and had been so traumatized that she was always fearful of attending medical appointments and of getting pregnant again. She said she did not experience any support for her situation in the UK.

The issue of health communication was raised, and whether explanations for medical procedures were clearly provided. Ghada described her experience:

They asked me a lot, explained a lot for me, why we should do that scan, we should do for that reason, for that reason, because if not we can't see the baby, and your er pregnant, your urine test, your blood test, you are pregnant, 7, 8 weeks, that's why like ask me (Ghada)

However, women's experiences varied from person to person. So, while Ghada seemed to be satisfied with her care, Soleen and Laila were less impressed, asserting that health practitioners could be patronising, and dismissive about their concerns.

Everything fine, fine, fine, take the paracetamol, I know to take the paracetamol. The midwife says but I know. (Laila)

In contrast, Ghada was encouraged to attend at the hospital for any sign that something could be wrong in her pregnancy. There was praise and appreciation for the midwife who cared for Nourhan as well as the health visitor who provided her with clothes and baby equipment.

my midwife she was really nice and lovely, yeah. She's really care about me, and she was really help me a lot, and you know when I give birth, she tell me, I am off today but I will just came for you, and she just came, and she help me to give birth, and when I open my eyes and see her, she was crying. She says you remember, I remember myself, yeah, in my first pregnancy. She was really lovely and nice.

When speaking about healthcare in more general terms, Maha complained about how long it took to book an appointment and to get her health condition seen to. She described having to wait a considerable time to be treated in hospital where she was not given a bed because the hospital was too busy. Maha also claimed she only became diabetic when she started living in the UK.

She said when I came to England, I wasn't have like any problem. I was really strong and normal. After I came here, you know there is no sunny, the weather is really bad, lost her friends, so, she have all these problems. The first thing she has was diabetes. (Nourhan for Maha)

Nourhan expressed a view that migration to the UK over time had caused Syrian women to suffer ill health whereas they were fit and healthy in their country.^4^

On the topic of healthcare preferences, most women would prefer a female doctor, or a male doctor with her husband or female nurse present. In an emergency however, anyone would do:

Nourhan: [Fatem] said if it is emergency she wouldn't care if it's male or female. When a woman is giving birth she would let anyone touch her to get the baby out. Even if it was a cow or what. Just get it out. You forget everything

The women also expressed different preferences about the gender of their health professional. Widowed ten years ago, Narin felt shy about a man performing a cervical examination on her. Nourhan was self-conscious about being fat, and embarrassed to be examined by a woman because of this. If a male doctor was present with a female nurse, Khalida would still feel uncomfortable. As for Maha, a male doctor examining her top half was fine, but not if it was her bottom half. Female rather than male interpreters were preferred at hospital appointments.

The women's accounts highlighted the importance of the doctor-patient relationship, whether in their countries of origin or in the UK. While they were used to paying for healthcare in their home countries, the women's experiences of healthcare in the UK being free at the point of delivery have also been mixed. Cultural beliefs regarding gender also affected their preferences about who should treat them, and how they should be treated and supported.

### 3.2 Pregnancy experiences

#### 3.2.1 Early motherhood and pregnancy loss

Most of the women started their families in their adolescent years, which was described as the norm. Maha expressed her discomfort at having an unplanned pregnancy as a teenager when she was not really prepared. The oldest of the women (Fatem) described marrying at 15 and having her first pregnancy at that age, which sadly ended in a miscarriage.

More accounts of miscarriage were then shared in the group. Altogether seven of the women had experiences of miscarriage, and some, understandably, were unwilling to recount much of their experiences in the group discussions. Several of the miscarriages occurred before the women arrived in the UK, or before they settled in the North East.

Nourhan's account was particularly detailed, as it occurred more recently in the UK, soon after her marriage at age 19. She explained that she had fallen at work hurting her back when she was 5–6 weeks into her pregnancy and had her condition checked at the hospital. A transvaginal ultrasound scan was performed, and she remained in hospital for seven hours. At seven weeks into her pregnancy, she experienced some bleeding and was unsure if it was anything to be concerned about. She explained:

I wake up and then I found blood like this [shows phone], I still got the picture, [like a clot]. I did the picture I don't know why, but when I'd seen the baby, it was really looking like a baby, I saw the eye, I saw everything, it was really like a surprise for me. And even when he was like look like baby, he wasn't have hand or leg but I don't know how I feel like this okay? It was only like 7 weeks. And also, I take it and put it in the bin, and I go to sleep, and I also felt I was still pregnant.

As the youngest participant, it was noted that she took a photo of the “clot” which she showed to her mother who immediately recognized it and asked her to go to the hospital where it was confirmed that she had lost the baby.

From what she related, it appears that there were several information gaps that were not addressed in her preparation for motherhood. Firstly, she was not aware that there could be some slight bleeding or spotting during pregnancy. Secondly, she was not alerted to the risk presented when experiencing heavy bleeding “like a period”. She had also not realised that taking a pregnancy test soon after a miscarriage might show a positive result, and that it was best to wait a few weeks.

A contrasting account was that of Ghada trying for a baby and becoming pregnant at age 36. She was overjoyed but then experienced some bleeding and underwent a transvaginal scan that provided reassurances. Unfortunately, two weeks later, at a scheduled ultrasound scan, they found that the foetus had died. This had a devastating impact on her, especially as it had to be managed medically to speed up the process.

they said the baby is not moving, the heart is stopped, your baby has died. I was in shock, crying, screaming, it was very hard. … it was 7 weeks when I had the miscarriage, and they gave me tablets for it to come away, comes out. I had the tablets, after two days, sharp pain, three o'clock in the morning when I was sleeping. …. they took me to hospital, and I stayed one night there, until the baby came out, and the worst feeling ever I had. …. I was crying all the time when I was seeing a baby or hearing a baby crying or laughing,

Like Ghada, Parveen recalled her experience of miscarriage, which was marked by heavy bleeding before and after the loss of the foetus and for an extended period of time.

I had bleeding, and like heavy bleeding, and I went to hospital, I had to stay in hospital for two days. They did scan, they said they can't see anything because of blood on your womb. And after that I had a miscarriage, I couldn't see anything, just bleeding, just bleeding, and I had bleeding for three months.

Ghada reported having a blood test every two weeks, to determine if she was still pregnant. She eventually resorted to her own contacts abroad to obtain medication which finally resolved her debilitating menorrhagia.

I was like oh I couldn't even work, I couldn't eat, just stay at home because of bleeding for three months. And after two months and a half I called my family.

The women went on to discuss what they viewed in the publicly available information online, regarding miscarriage. They found this helpful for example the opportunity to learn about the different ways that the products of conception could be safely evacuated from the mother's womb. What these women's accounts highlighted were the severe consequences of having a miscarriage, how better information could have prepared them for the experience, and how they would resort to their overseas networks if they felt let down by maternity provision in the UK.

#### 3.2.2 Scans and prenatal diagnosis

Discussions about prenatal diagnosis and the use of ultrasound scans drew out interesting issues and concerns, as well as comparisons between the women. They were familiar with antenatal screening such as for the detection of Down Syndrome and the test for gestational diabetes. The anxiety experienced by women about the possibility of having a baby with a disability was expressed in the following:

My friend has same problem, they told her, baby [·] Down's, 50/50 chance, and she was crying, crying, and then the baby was normal. (Parveen)

Because of not wanting to face that level of uncertainty, and to avoid unnecessary stress, Parveen refused the test for Down Syndrome. As Fatem responded, “Make too much headache”. While Parveen felt able to call on the emergency services to help her when she was in pain, she did not agree to a CT scan for her breathlessness, worried it would affect the foetus.

Despite these anxieties, the women were comfortable with ultrasound scans to reveal the viability of a pregnancy. Khalida described an occasion in Syria when a scan was able to discover an anomaly in her daughter's pregnancy, which prepared them for the miscarriage.

However, one issue that the women discussed at some length was that of the transvaginal or transabdominal scan, which was described as quite invasive in this account:

I come by myself, and she tell me, can you please take your pants off and your underwear. I said, what? Whereas I was really scared, and then I see this is a long scan, and I goes please tell me this is not for me. Tell me this is not for me. She tell me no this is for you. What? You want to put this inside me? If you don't like, it's okay, it's for you and it's really important, and she just let me do it, it's important for you to find baby. (Nourhan)

So, when she did the scan, I felt little bit pain, I told her, this is hurting me, and she said, okay sorry I think we stop. I think she was a “learner”. (Nourhan)

Following this experience, there was the miscarriage, which Nourhan attributed to the transducer or probe entering her vagina. She went online and discovered that women of Middle Eastern heritage had reported miscarriages following the use of the scanning device. It was explained to her at the workshop that the scan was used specifically in the case of pregnancies of 7 weeks and below because of the size of the foetus and the risks involved at such an early gestational age. The community midwife who was invited to attend one of the workshops, also provided reassurances of the safety of that procedure but doubts remained.

The question of consent for the scan arose when the other women joined in with their experiences. In Parveen's case, the foetus could not be seen on the ultrasound because of the presence of a lot of blood in the womb, and consent for the scan was clearly explained. However, Parveen's pregnancy was over 8 weeks, and because her first ended in a miscarriage, the health professionals appeared to exercise more caution. But in Nourhan's case, informed consent apparently was not sought. This experience caused her to turn down the offer of the transvaginal scan for her second pregnancy, despite her mother's encouragement. Several explanations were offered for Nourhan's experience, such as the sonographer who needed to have had more supervision.

While most of the women were only too ready for the birth of the baby after having carried it for so many months, Ghada wanted to “keep her daughter forever in her tummy” (Interpreter for Ghada).

Yeah, I was pregnant. I don't want to leave my baby. I want to just keep it inside me. I swear to God, my husband was saying, you crazy. See, I don't want her to be around anywhere except my tummy.—(Ghada)

This could be explained in terms of the security of having the child safe and close to her, with the assurances provided by the prenatal scans especially after her trauma of a miscarriage.

Another issue of contention arose with respect to scans being able to indicate if the baby was male or female. Nourhan was indignant when she was reprimanded (perhaps jocularly) for wanting to know the gender of her first child.

Just check for me if it's a boy or girl, she tell me it doesn't matter if it's a boy or girl. The problem just like the pink color. … And then she tell me, I can't see well. And then I just go for private scan, and they tell me it's a boy … she was really like, annoying, why she tell me like why you care if it's boy or girl like, it's my son like, it's my child, like.

She was not the only mother who resorted to having a private scan. Ghada also had a private scan, costing £70, which to her delight confirmed that her baby would be a girl. The women quoted cases where fertility specialists enabled their relatives to choose their baby's sex.

Informed consent is an essential part of medical care which prioritises patient autonomy in health decisions, regardless of their background characteristics. Nevertheless, health beliefs can vary among patients, which can be influenced by social media, personal and cultural values, affecting their decision making about antenatal screening and prenatal diagnosis. In maternity care, it is important to respect migrant mothers' beliefs, values and preferences in their pregnancy choices, but the accounts given by participants show how this can be lacking.

#### 3.2.3 Home remedies and self-reliance

Most of the women described symptoms of nausea and vomiting that led to weight loss. This included their experiences in their own countries, where forms of clinical support available included nutritional supplements. For two women, an extended period of hyperemesis applied to all their pregnancies. To ease nausea and vomiting, customary foods were recommended, such as lemon, cucumber, spicy pepper or chilli, and iced water. The women found it helpful listening to advice from one another about how to manage their pregnancies. The two topics they were most interested in were giving birth and feeding their new-born. The discussion also included useful information about how to care for the stretch marks that would develop, with women recommending bio-oil, shea butter, or coconut oil.

When the women were asked if there were any traditional ways to help ease the birth of the baby, different health remedies were offered. These ranged from mint effusions to pineapple when the baby was overdue, and cinnamon, or halva—a traditional sweet—or dates to be eaten to help the recovery.

my uncle's wife, I saw her experience that's why I did it again, you know mint, fresh mint, she put in the hot water, she just put here and she was sitting like this, and cover herself, and you know all the, it's hot, and the steam goes into the vagina, and makes it more space, so the baby comes out? So, it's easier. (Ghada)

While the experience of a forceps delivery was appreciated by Soleen, that of gas and air was uncomfortable for Nourhan, because it prevented her from being able to express herself.

she give me this gas. I hated this gas—no it was really bad, because when I was taking this gas, I was, can't shouting—I want to speak like, I can't speak at all

Another concern for the women was how to take care of their new-born baby. Most of the women were able to breastfeed and shared their experiences. One helpful piece of advice was the following:

During the pregnancy the last month, you have to do shower, hot shower, with Vaseline, for one month, to get yourself ready for the baby. She said when you did this, you will not have any like bleeding. Do some massage, this will prepare yourself (Interpreter for Maha)

This advice was given in response to one of the mothers who experienced bleeding nipples. A mildly alcoholic aniseed drink for helping the baby to sleep was also recommended.

Amidst these home remedies, there were several instances throughout the workshops where the women's self-reliance and strength were referred to as positive personal characteristics. There was a degree of pride in one's ability to withstand the discomforts of pregnancy and childbirth. Healthcare professionals were also described as encouraging the women to draw on their strength:

They said, don't worry, you'll be fine, you're strong, you know. So, thank God my baby's three years now. (Ghada)

The doctor told her, you are beautiful and strong. (Interpreter for Narin)

Nevertheless, the women recalled going through periods of deep distress:

Yes, I had, I was crying every day, especially around 5, 6 in the evening time [me too; me too]. Help? No, I had no help because I didn't ask for help [me, too]. But I was crying all the time (Ghada)

While many women appeared to cope on their own with their perinatal mental health, Parveen was able to access help from her midwife, who referred her to a counsellor.

These accounts pointed to the benefits afforded by natural remedies that the women were accustomed to using, and the importance of personal strength and resilience. Everyone agreed that experiences of pregnancy can vary depending on the mother and the pregnancy that was carried. In recognizing individual differences, the women recognised that there was a need to take responsibility in understanding one's own body, to draw on personal experience, and not to take advice from others too eagerly without reference to one's own sense of self. This insight was drawn from the body mapping exercise that the women participated in.

### 3.3 Information and communication

#### 3.3.1 Language, information and support seeking

While most of the women were literate in Arabic, there were two who were not; Parveen, coming from Iran, could not understand Arabic, and Maha was not able to read and write Arabic. To overcome this, English was used, which Parveen was fluent in, and Nourhan did most of the interpretation for Maha, her good friend. When asked if they would like the co-created resource translated to Kurdish, Ghada explained that she only knows Kurdish “slang” because she was disallowed from using Kurdish in Iraq. However, she recalled reading some books in Kurdish at home as a child. Parveen was invited to translate the ERicar2 resource into Kurdish as she was fluent in it. Some antenatal health information that was shown to the women had been translated into Kurdish language. In viewing the translated material online, the women noted that some documents were incorrectly formatted left to right instead of right to left.

When asked about the resource being in English, Nourhan felt that it needed to be in English that was easy-to-read. She drew the distinction between the younger and older generations of Arabic-speaking migrants. According to her, those under 30 would be able to manage with their limited English and Google Translate. For Laila, it would be better if they were able to improve their English in classes and not have to depend on an interpreter particularly in discussing intimate details.

The extent to which apps such as Google Translate is used in clinical consultations is unknown, but with the advent of AI, is likely to be increasing. Among the women, none of them were able to share any experience of on-demand interpreting services such as LanguageLine or interpreters over the phone. The women were usually accompanied by their English-speaking husbands or otherwise had to depend on different interpreters each time.

There was a general feeling that when it came to information, convenience and accessibility were most important to the women, although other factors may come into play e.g., level of education, experience of pregnancy. Regarding written materials in the form of a book, there were responses indicating the preference for reading information online because “it's much easier”. Women like Fatem literate in their own language described reading information in the form of a book.

While some women accessed information from online communities, antenatal classes as sources of information were not accessed by the women because they were unable to attend, for e.g., because of being at work, or because their pregnancies occurred during Covid. Personal and direct communication with professionals was much valued by women.

Seeking help directly from professionals was also recommended and the relationship with their midwife was key: “my midwife, like best friend, and she told me, feel free to text me send me message all the time about everything”. The need for support for their mental health seemed particularly important because of the rates of miscarriage observed among them when they related their pregnancy experiences. Attendance at appointments was regarded an important means of obtaining information and support, and the women had different strategies to help them keep their appointments e.g., calendars and alarms that could be physical or digital.

Social media was widely used by the women in the group. They all belonged to the “Tavga” WhatsApp group. Nourhan used Google regularly, while Fatem claimed that there were numerous YouTube videos in Arabic that she could access for health information. Nourhan described how she obtained information about the risks of the transvaginal scan from TikTok:

they said, please tell all of them stop doing this, even with all women … But little bit of them, like 2% 10%, they were said, no, it was alright, fine. But like majority of them they were saying it's really bad.

When asked who used Instagram, Facebook or Tiktok, Nabiha responded by saying that she thought they all did. There was widespread use of AI and the women mentioned SnapChat, ChatGPT, MyAI, Blueface, with comments such as “I use it for everything”, and “this one is like a friend”. Parveen commented that AI was helpful when she was in college, and it was most useful when writing formal letters.

Laila and Nourhan were very positive about pregnancy apps and mentioned two in English, i.e., Flo and BabyTrack, while Soleen has used one in Arabic, called “Pregnancy Journey”. Laila would recommend the Flo app to Dila, the fictional character, explaining all the benefits, such as answers to questions, reading about others' experiences, reviews, and the sharing function. Nourhan was able to point out the functionalities available in a pregnancy app, which included language support and the community function where help was easily available.

Soleen brought up useful aspects of social media as she used both Facebook and Instagram and drew on them for information around childrearing. While there could be some useful information from searches online, Parveen described some of the possible challenges, as there could be inconsistencies in the different translations, and possibly conflicting information to deal with.

it is difficult, to use the app, to translate, (even if the app is in Arabic) yes, it will be difficult because when you read in Arabic, you have to speak with your GP or midwife in English, and when she explain something for you in English, still difficult for you. Arabic says that, Kurdish says that, and English say like different.

The question was put to them about how they were able to trust the information on these sites. Nourhan for example drew on her intuition and cross-checking with reliable sources of information from the NHS. In deciding on what could be trusted, the women would consider the reviews and the stars given for different providers, but Soleen would judge the information based on what she thought was “good for her”—specifically. While she and Ghada would check the reviews and pick the best one, Nourhan noted that reviews could be fake and written to win customers. Laila preferred to ask her midwife to recommend online information rather than to search on Google. With all the information to take on board, which can be confusing, women felt it was important to trust one's own instincts and not be overly anxious.

Communication on social media can be fraught especially with the current situation in the Middle East. The women spoke of exchanges online where they were badly affected by upsetting personal comments: “It was like broken my heart, …. – I just deleted the message”. While the women were quite au fait with how to handle difficult situations online, it could still have an emotional impact on them. While the women were ardent users of social media, they were more wary when it came to internet sites that the fictional character Dila should visit, recommending the more reliable sites especially NHS, and NHS endorsed information sources. This was because of her unfamiliarity with the UK and how information could also vary from country to country.

Thus, the women, with their different language abilities, drew on a range of personal and digital communication as well as sources of information to form their support systems during pregnancy. While Google seemed to be the default resource for several women, YouTube was quoted as a plentiful source of advice, with the advantage that the videos were available in Arabic language and those who were semi-literate could obtain the information they needed. Pregnancy apps provide a combination of personal and impersonal features and were looked upon favourably by the women. But support from health professionals and NHS endorsed sites were felt to be the most reliable and dependable sources of information.

#### 3.3.2 Identifying key messages through co-creation

In co-creating the antenatal care information resource, it was agreed that there should not be too much information, but a focus on key messages. The women were presented with ideas about the resource: line drawings could be accompanied by tips, messages, strategies, and these could be for Dila, building on the advice from the group. The women could also create a little story with messages and advice, in the form of a booklet, translated into Arabic and even Kurdish. CC shared the Roma zine^5^ and how it was constructed, with QR codes allowing women access to trusted information sites. They were also introduced to collage-making, used in ERicar. An idea from Narin was to create a big cartoon of a pregnant woman and each of them contributing comments or advice with their names attached. Another was women contributing pictures or cartoons or handicraft, which would be printed and cut out. The women also had the option of doing the drawing or collage themselves and helped in putting it all together.

Messages for the fictional character Dila as a new migrant mother were elicited from each of the women through their artwork and one-to-one discussions. Ghada and Nourhan for example, were clear about Dila having to pay attention to her own needs and wants above all else. While the women agreed that they had to draw on their own strength to cope, they also spoke of the help of God to do this. These beliefs were reflected in their messages to Dila, who they encouraged to “stay strong” and to think positively about how it was going to end. But women like Dila had to be encouraged to take the initiative to explore the help available, with the workshop on antenatal care resources (workshop 6, Table 1) able to signpost women to support online. While the exhortation to “stay strong” was important, there could also be dangers to one's mental health, in keeping to oneself all the time, as pointed out by Parveen who focused on the benefits of education. Nourhan was concerned that Dila should not ignore the small changes in her body as they could be warning signs. Among all the women, Laila was the most emphatic in encouraging Dila to access all the available antenatal care services and support networks available, for both her physical and mental health and wellbeing. Her comments about midwives were encouraging:

Also midwife, they are very good, they can help you more than, more than mother, in my experience, more than your mother, more than your friend or your sister. Because they really answer all the questions.

Other comments included the importance of meeting friends socially and accessing community groups especially as Soleen described how she was embarrassed about going out on her own with a baby crying but found a family hub helpful. English classes were also recommended, as Laila pointed out that patients needed to also understand written information. The women were also able to signpost Dila to English classes, and charities providing free lessons. The possibility of having spoken information such as in a video was also discussed. Other recommendations included vitamins and healthy food, avoidance of runny eggs and advice to read the Koran and to pray as part of their self-care practice.

The importance of digital and physical images in conveying information was expressed through women bringing in their own illustrations to add to materials provided for the collage making. The collated result was presented to the women, written in the form of a letter that drew on the most pertinent messages that the women had shared. As an exercise in validation, the letter was read in English and Arabic, with opportunities for the women to respond to what was written.

When the letter to Dila was presented, there were strong reactions to the dilemma that Dila had of finding out the baby was going to be a girl when a boy was preferred:

just tell him the girl is really nice, she's like really love her family, and she will be having a really beautiful child; she will be like so close to you, more than a boy [like a queen]. And then he will know like the girl is going to be like the most nice thing in her mind [more beautiful than the boy]. (Interpreter for Narin)

The recommendation for dancing as one of the favourite activities in their community being a form of physical exercise was debated. It was decided that it was left out because of the risk of too much strenuous exercise during pregnancy. The women felt it was important to include preparation for emotional changes during pregnancy, likely due to hormonal changes. However, it needed to be stated that every pregnancy is different.

The women reacted positively to the letter: “really good and easy to read, like for me, it felt really nice. I like how you cut it”. They were asked whether it should be in the form of a greeting card, whether there should be more obvious Arabic styles of writing, two different letters to cover more topics, or one for the health professional to pass to the patient as a means of support. The last option was the agreed format, and the letter was signed off with three names but eventually there was agreement for all their names to be on the letter.

To summarise, the co-creation work in our previous project (ERicar) formed the basis of activities with the Arabic-speaking women, but in ERicar2 there were more contributions of illustrations and hand written notes. Their messages were similarly focused on Dila's need for self-care, positive thinking, and accessing support but included equipping oneself with language skills and information and knowledge through online resources. Additionally, Dila should be confident and self-assertive especially if faced with traditional attitudes about the baby's gender.

## 4 Co-creation processes and the prototype

One of the strengths of the project was our longitudinal engagement with the women, which took place through ten workshops and one debriefing session. Co-creation was scaffolded by a number of hands-on and visual-based methods, used to support storytelling and interpretation over time. Building on ERicar through the fictional character Dila as a newly arrived pregnant Arabic-speaking woman helped elicit responses from participants on themes or stories. Women were prompted to think from the perspective of Dila in different scenarios; for instance, how she might use digital technologies (i.e., pregnancy app) and what support networks she would need or have during her pregnancy journey (Figures 2, 3). Using Dila as a discussion point worked well for women who were asked whether they could identify with her, prompting them to reflect on their own experience and compare it with the character's narrative. Activities in small groups worked particularly well especially for participants with language barriers: women worked in pairs or groups of three where at least one woman in each group was confident with translating for others if needed.

There was no pre-conceived idea of the “final” resource we wanted to co-create with the women. In workshop 8, we showed participants examples of resources including the zine co-created with Roma women in ERicar. Women were generally positive towards the different examples and whilst this helped them consider key messages they would like to communicate; it was difficult to identify a particular format for the final co-created resource.

**Figure 5:**
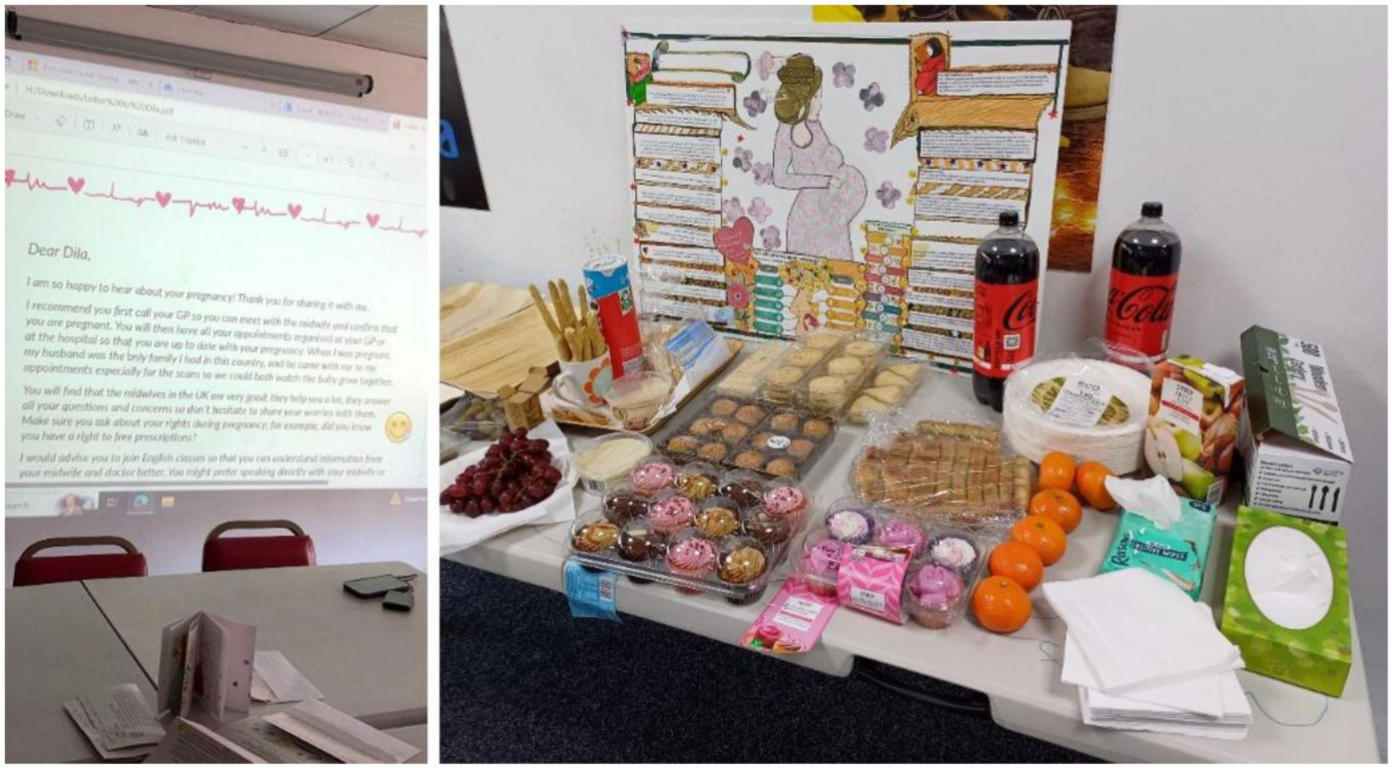
Feedback on the resource prototype “letter to Dila” in workshop 10 (left). Debrief and celebration in final session.

This made us think of alternatives such as a letter that the women could write to Dila. In workshop 9 the key messages that women wanted to include were refined by women writing notes, making a collage or drawing. Messages were compiled and arranged into a narrative to form the “letter to Dila” which was showcased in the last workshop (Figure 5 below). Feedback was collated on what should be changed, added or removed and the potential of having an audio-visual version of the letter discussed, to cater to different needs. Our advisory panel reviewed the letter and provided recommendations, in particular the importance of taking folic acid early on in pregnancy and finding out about vaccinations.

We revised the content of the letter to address all the feedback and collaborated with a department colleague who helped us bring the letter to life by creating an animation. Part of this process included developing a storyboard, creating new visual content inspired by the workshops' contributions and inviting two of the participants to our lab to audio-record the content of the letter in both English and Arabic. At a debriefing and celebration session, we projected the animation before inviting participants to reflect on the outputs and their wider experience of taking part in the project. Feedback from 21 women from the Tavga community, and a group of nine North African Arabic-speakers in a separate community hub was positive overall. The resource has been promoted to health providers, with project outputs made available online, hosted by OpenLab, Newcastle University^6^.

**Figure 6:**
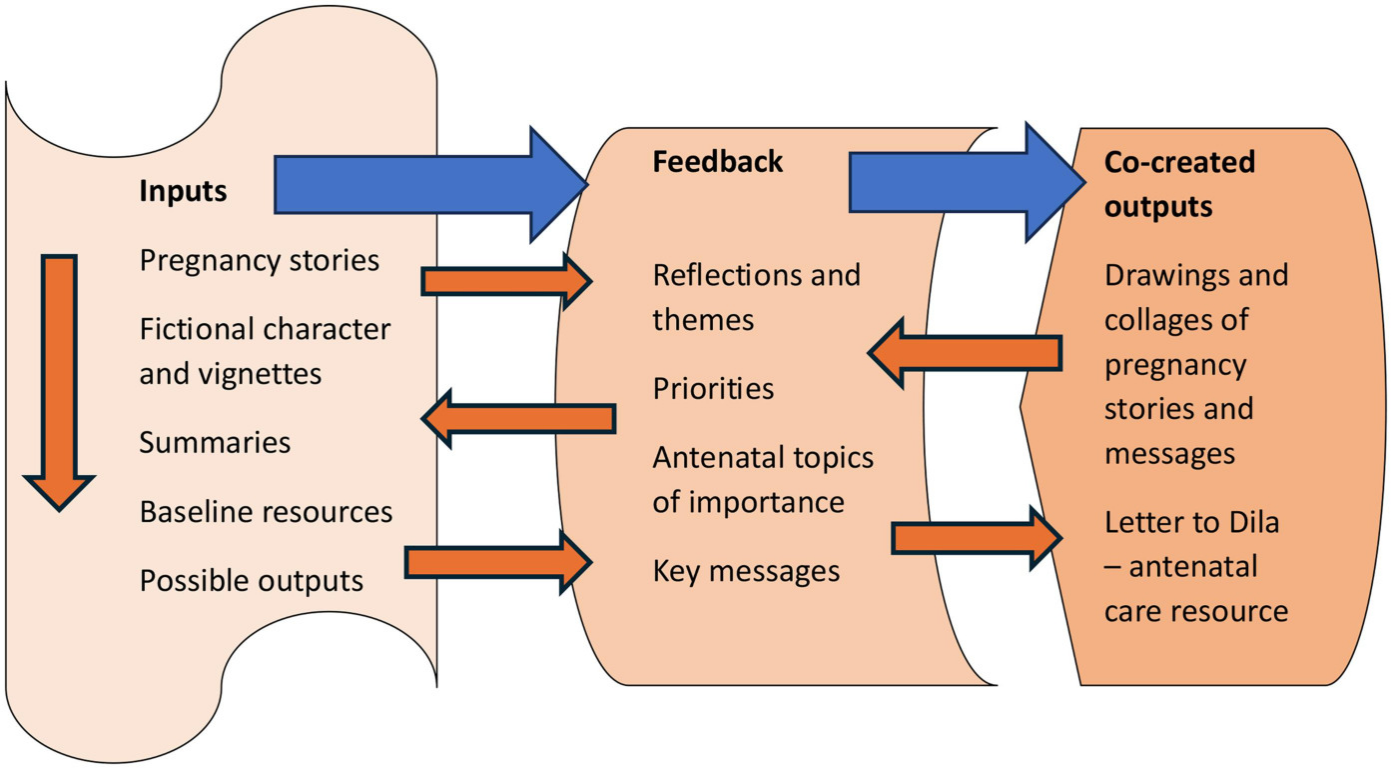
Diagram of iterative inputs and co-created outputs.

To summarise, our approach to co-creation can be described as a form of “research bricolage” (31, 32) in which researchers act like “bricoleurs” or craftsmen. In our case, it meant being flexible with how we planned our workshops, and responsive from one session to the next. With this approach, we did not start with an idea of what to create but we used examples to guide and inspire participants and integrated their preferences and ideas as we progressed through the workshops. The bricolage approach meant that we had to use materials and resources at hand and a colleague who had expertise in animation software was persuaded to help us develop the project's final output (the animation of the “letter to Dila”). The iterative co-creative methods used in the project, as presented diagrammatically (Figure 6) below, can be replicated, or tailored for use in other minority groups.

## 5 Discussion

### 5.1 Study limitations, challenges and opportunities

As in the work with the Roma in ERicar, the ERicar2 study was limited in having a small number of women not entirely representative of the community in the North East, and none who had arrived after 2019. In addition, there were only a few women with more recent experiences of childbirth. The creation of Dila as the fictional character while led by researchers was nevertheless drawn from the women's narratives and fully endorsed by participants. With less constraints on time, the prototype could have been more directly driven by the women.

Language and communication posed challenges, for e.g., women who were not literate in Arabic. Women in the group fluent in English were more assertive than the interpreter and eventually took over when she left the project, and this had to be carefully managed. Such difficulties arising in group dynamics is to be found in many similar contexts (33, 34), and attending to the quiet conversations on the sidelines provided some important background insights to be carefully considered in our interactions with the mothers.

As the workshops progressed, different voices became more prominent as confidence grew, especially when they observed that non-standard conversational English was acceptable. Others were content to listen and learn English only offering their perspectives when asked directly. Some women enjoyed speaking more than creating artwork, those who were literate in English were able to express themselves in words more than in illustrations, while others contributed impressive artwork. Additional time was needed for more creative aspects following the workshops, in particular the idea to develop a digital version of the “letter to Dila”.

### 5.2 Engaging with migrant mothers

Underestimating the importance of how cultural differences impact the lives of mothers can be a hindrance to research engagement with minority ethnic groups. Women's involvement in research may be contingent on their relationship with their partners, such as agreement needing to be sought before women could participate. Assumptions about women's autonomy had to be revised, but most of all, trust in the gatekeepers was required for recruitment to be successful. In research planning, time and effort in recruiting from marginalized groups need to be considered. Interpretation and translation are complex processes because of the variety of languages and the levels of fluency involved. We found that flexibility in being able to navigate these communicative challenges using a mixture of media was helpful, e.g., spontaneous use of photos from mobile phones, Google Translate, YouTube, physical line drawings and pictures—assisted in making meaning clear. Multiple interpreting by different people in the group was also a way of meaning-making and the consolidation of understanding. Aural readings of text aided those for whom the written word was less accessible. All these activities were carried out in an environment in which the women were relaxed and comfortable enough to share together in what could be described as a safe space within rules of engagement. The concepts of bricolage and iterative co-creation were useful frameworks for reflecting on and undergirding these processes.

From feedback during the debrief and celebration session, what women gained from the workshops included the opportunity to meet and share experiences, while recalling their pregnancy journeys. For some, it was a chance to improve their English. Hearing others' experiences helped some appreciate their care, while others benefitted from the advice provided by more experienced mothers. Information about the maternity exemption certificate alerted women to their rights, and the meeting with the community midwife provided reassurances. Older mothers discovered information that would be helpful for their daughters and daughters-in-law. Women thought the “letter to Dila” was more personal than a leaflet, just the right length, and easily shared with other women. They agreed to include their signatures to the letter, as an indication of their combined effort in co-creating the resource.

### 5.3 Implications for healthcare providers

Many of the recommendations in ERicar^7^ applied to ERicar2, for example, the importance of the relationship between midwife and mother, and the continuity of that relationship. This is especially so with the loneliness and lack of family support reported and the impact on their mental wellbeing (11). The number of miscarriages reported appeared linked to the high incidence of adolescent pregnancies in this group (35). These experiences of pregnancy loss suggest the need for more awareness, preparedness and support, including properly updated maternity records. Support needs for Muslim women experiencing miscarriage have been highlighted in previous research (36). Consent for the transvaginal scan was also a point of concern, particularly explaining the risks associated with the procedure. This case resonated with the need for knowledge claims to be negotiated with culturally trained health professionals (16). The request about finding out the sex of the baby showed the need for cultural sensitivity. Similar indications of clinicians being patronizing towards women echoed reports of paternalistic attitudes and assumptions about women's identities (12, 13).

The mothers shared natural remedies that could be helpful especially nearing the time of birth. However, as studies have suggested (14), and the workshops demonstrated, counselling on evidence-based medical interventions would benefit women from this community. Because of their preference for privacy and confidentiality (13), the importance of learning English was raised. When faced with systemic barriers and a lack of care, women were found to resort to advice online and their own family resources abroad. This pointed to women's agency but also gaps that could be filled in NHS care and information provision, such as via intercultural interpreting services (37). Nevertheless, there was also evidence of exemplary care from midwives that was reported and much appreciated.

## 6 Conclusion

Despite the study's limitations and challenges, the replication of ERicar in ERicar2 among Arabic-speaking migrant mothers proved to be a feasible endeavour. The qualitative findings and lessons learnt in research terms, intellectual understanding and health implications are wide-ranging. Essentially, understanding resettled migrant mothers requires the community connections, which in this case were developed through relationships of trust and respect previously established. These facilitated recruitment, the smooth running of workshops, and the open and friendly interactions within a safe community space. Being flexible and “making do” as in a “bricolage approach” as well as the iterative checking in with participants regarding their views were key features in the workshops. Taken together, these were the ingredients for the co-creation processes to work well, and the resource to be developed based on the honest sharing of the women's personal lived experiences. Insights gained appeared to support existing research about systemic barriers such as paternalism and difficulties with interpreters, but new knowledge included women's agency in the use of natural remedies and transnational information networks. The role of religion in providing a supportive belief system was also included in women's accounts. Considering the various digital channels that the women were regularly using, there is scope for development in the subject area of Human-Computer Interaction, to consider the benefit of more personalized less medicalized forms of health promotion and communication (19, 20, 23). The role of AI for example in language translation, and the importance of challenging stereotypes in digital representations of ethnically marginalized groups are some of the research areas that could be pursued.

## Data Availability

The original contributions presented in the study are included in the article/Supplementary Material. Further inquiries can be directed to the corresponding author.

## Footnotes

^1^QuillBot Translate: Accurate AI Translation in 50+ Languages was used and the translation was checked by the community coordinator and the interpreter.

^2^ https://vimeo.com/1094007755

^3^Southport trial: Knife attacker pleads guilty to murdering three girls - BBC News.

^4^This appears to support the ‘healthy migrant hypothesis’ or ‘healthy immigrant effect’ (Kennedy, Kidd et al. 2015) where it was observed that immigrants had better health than native-born residents, due to selectivity i.e. the healthiest being able to leave their countries.

^5^ERicar zine - ePrints - Newcastle University.

^6^ERicar2: Engaging Resettled migrant women in the co-creation of an antenatal care information resource | Open Lab.

^7^ERicar: Engaging Roma women in the co-creation of an antenatal care information resource - ePrints - Newcastle University.
